# Reactivation of Recall-Induced Neurons in the Infralimbic Cortex and the Basolateral Amygdala After Remote Fear Memory Attenuation

**DOI:** 10.3389/fnmol.2019.00070

**Published:** 2019-04-17

**Authors:** Ossama Khalaf, Johannes Gräff

**Affiliations:** Laboratory of Neuroepigenetics, Brain Mind Institute, School of Life Sciences, École Polytechnique Fédérale de Lausanne (EPFL), Lausanne, Switzerland

**Keywords:** engram, basolateral amydala, infralimbic cortex, fear extinction, reconsolidation, remote memory, updating, memory trace

## Abstract

Whether the attenuation of traumatic memories is mediated through the suppression of the original memory trace of fear by a new memory trace of safety, or through an updating of the original fear trace towards safety has been a long-standing question at the interface of neuroscience and psychology. This matter is of particular importance for remote fear memories as they lie at the core of stress- and anxiety-related disorders. Recently, we have found that in the dentate gyrus, the effective attenuation of remote fear memories is accompanied by a reactivation of memory recall-induced neurons and that the continued activity of these neurons is critical for fear reduction. However, whether this also applies to other brain areas implicated in the storage of remote fear memories remains to be determined. Here, we show—by cellular compartment analysis of temporal activity using fluorescence *in situ* hybridization—that such reactivation also occurs in the basolateral amygdala and the infralimbic cortex, two brain areas known to be involved in fear memory attenuation. These results provide further experimental support for effective traumatic memory attenuation likely being mediated by an updating of the original fear trace towards safety.

## Introduction

Post-traumatic stress and other anxiety disorders range among the most enduring forms of memories. Remembrances of traumata months later in rodents (Debiec et al., 2002; Frankland et al., 2006) and years after the original insult in humans are commonplace (Ringburg et al., 2011; Haagsma et al., 2012). The lifetime prevalence of post-traumatic stress disorder (PTSD) in the general population is estimated at 7% (Kessler et al., 2005), and this number at least quadruples among individuals having suffered severe traumata such as war or sexual assault (Davidson et al., 2004; Javidi and Yadollahie, 2012). Because of the persistent nature of traumatic memories, early interventions are considered of prime importance (Davidson et al., 2004; Kearns et al., 2012). Yet, such interventions are oftentimes not readily available, which places a strong emphasis on better understanding treatment approaches for remote traumata (McCleery and Harvey, 2004; Centonze et al., 2005).

Among the most effective treatments for traumatic memories are exposure-based therapies (Foa and Kozak, 1986; Foa, 2000). In these therapies, patients are repeatedly confronted with the trauma-eliciting stimulus in a safe environment, with the premise that the fear associated with this stimulus will eventually subside. On the one hand, such repetitive re-exposure is thought to induce the formation of a new memory trace of safety, one that suppresses the original memory trace of fear, and thus leads to the extinction of the original fear memory (Bouton, 2004; Myers and Davis, 2007; Quirk and Mueller, 2008; Pape and Pare, 2010). Indeed, several studies have shown that during memory extinction of an aversive tone, a different set of neuronal subpopulations in the basolateral nucleus of the amygdala (BLA) is recruited than for its initial memory formation (Herry et al., 2008; Ehrlich et al., 2009; Trouche et al., 2013). Such suppression is likely to be mediated by inhibitory circuits projecting from the infralimbic (IL) region of the prefrontal cortex, whereas the expression of the original fear memory was shown to depend on excitatory projections from the prelimbic (PL) area of the prefrontal cortex (Herry et al., 2008; Ehrlich et al., 2009; Maren et al., 2013).

On the other hand, the cellular mechanisms of exposure-based therapies may also be mediated by a process referred to as reconsolidation-updating (Tronson and Taylor, 2007; Monfils et al., 2009; Nader and Hardt, 2009; Schiller et al., 2010; Clem and Schiller, 2016). Each time a memory is being recalled, it enters a period of lability (Misanin et al., 1968; Nader et al., 2000), the so-called reconsolidation window. This time-limited window is thought to allow the reactivated memory to incorporate new information pertinent to the present environmental contingencies that might no longer be the same as at the time of encoding (Dudai, 2006; Hupbach et al., 2007; Tronson and Taylor, 2007; Lee, 2008; Nader and Hardt, 2009; McKenzie and Eichenbaum, 2011). Thereby, memory reconsolidation helps the memory to be either maintained—when similar situations are encountered at learning and recall, strengthened—when a higher valence is encountered at recall, or weakened—when a lower valence is encountered at recall (Sandrini et al., 2015; Clem and Schiller, 2016). This third scenario is ideally suited to incorporate safe or non fear-eliciting information into a fearful memory trace so that its fear component is updated towards one of safety and no longer persists in its original form (Parsons and Ressler, 2013; Sandrini et al., 2015).

Surprisingly, the vast majority of studies aimed at deciphering extinction from reconsolidation-updating processes have been conducted for 1 day-old fear memories, leaving it unclear which of these mechanisms takes place when remote fear memories are being attenuated. Recently, we showed that reconsolidation-updating mechanisms are critically involved in the attenuation of remote traumatic memories (Khalaf et al., 2018). Focusing on the hippocampus because of its documented re-engagement upon remote memory recall (Debiec et al., 2002; Goshen et al., 2011; Gräff et al., 2014), we demonstrated that the reactivation of recall-induced neurons in the dentate gyrus (DG) not only accompanied behavioral attenuation of a 4-week-old fear memory, but that the continued activity of recall-induced neurons is necessary for memory attenuation (Khalaf et al., 2018).

Notwithstanding, whether similar processes also occur in other brain areas remains unexplored. Given the more distributed nature of remote contextual fear memory storage, which involves areas of the prefrontal cortex as well as of the amygdala (Frankland and Bontempi, 2005; Wheeler et al., 2013; Kitamura et al., 2017; Albo and Gräff, 2018; Silva et al., 2019; Zhou et al., 2018), this question is of considerable interest.

## Materials and Methods

### Animals

Wild-type C57BL/6 male mice were used. All mice had food and water *ad libitum*. Mice were at least 10–12 week old at the start of the experiments. All animal experimentations were done and approved under the cantonal veterinary authority in Switzerland (VD2808 and VD2808.1).

### Behavioral Paradigms

#### Contextual Fear Conditioning (CFC)

Animals were acclimatized for 2 days to handling several times a day. Contextual fear conditioning (CFC) training consisted of a 3-min habituation of the mice to the conditioning chamber (TSE systems) followed by three 2 s foot shocks (0.8 mA) with an intertrial interval of 28 s. After the shocks, the animals remained in the chamber for an additional 15 s. Three weeks later (spent in the home cage, during which animals were monitored for their overall health), the massed fear extinction paradigm was carried out. The home cage control group consisted of mice being exposed to the CFC, but without a recall nor the massed extinction session.

#### Massed Extinction

Animals were re-exposed to the conditioning chamber for 3 min without receiving the foot shock (to recall the memory), and returned to their home cage for 45 min, after which they were once again exposed to the training chamber for a total of 18 min. Extinction memory (EM) was tested by a 3-min context exposure 24 h after the last extinction trial.

### Cellular Compartment Analysis of Temporal Activity by Fluorescence *in situ* Hybridization (catFISH)

C57Bl6/J mice were contextually fear conditioned and tested for the memory 21 days later. Forty-five minutes following the recall session, the animals underwent the massed extinction paradigm, after which they were sacrificed at specific timepoints by cervical dislocation, and their brains were extracted and snap frozen directly using isopentane and dry-ice. For the home cage control group, animals were taken out of their home cage and sacrificed straightaway. All brains were cut with a cryostat (CM3050S, Leica Biosystems), and the coronal slices (20 μm) were attached on charged super frost plus slides (Thermo Fisher). The *in situ* hybridization was carried out at the EPFL histology core facility following the manufacturer’s protocol of the RNA probes (RNAscope, ACDBio). Two RNA probes were used against the immediate early gene (IEG) markers Homer1a (H1a), and cFos. The H1a probe was conjugated with Alexa Flour488 fluorophore, whereas the cFos probe was conjugated with Atto550. For each slide used for catFISH, an internal control was performed to detect the housekeeping gene Ppib and the bacterial gene Dapb, which served as positive and negative control, respectively.

### Image Acquisition and Quantification

Images were acquired using a Zeiss LSM700 laser scanning confocal microscope. Four different brain slices from different animals [six for the conditioned stimulus (CS)-unconditioned stimulus (US), and four for the home cage control, respectively] were quantified. The images were acquired with a frame size of 1,024 × 1,024 pixels using tiling mode and a 40× oil-immersion objective to achieve the highest resolution. The cells were counted with the cell counter plugin of Fiji. H1a and cFos positive cells were quantified in their corresponding separate channels, and then both channels were overlapped with their markers to identify the double positive population. The rates were calculated according to the formulas below.

$$Activation\;Rate=\left(\frac{cytoplasmic\;H1a+cells}{Hoechst+cells}\right)\times 100$$

$$Learning\;Rate=\left(\frac{nuclear\;cFos+cells}{Hoechst+cells}\right)\times 100$$

Reactivation Rate=(cytoplasmic H1a/nuclear cFos+cellscytoplasmic H1a+cells)×100

### Statistics

Statistical analysis was done using Prism 6.0 (Graph Pad) as described in the figure legends. All *t*-tests were two-tailed unless otherwise indicated, and the level of significance (alpha) was set at *p* < 0.05.

## Results

**Figure 1:**
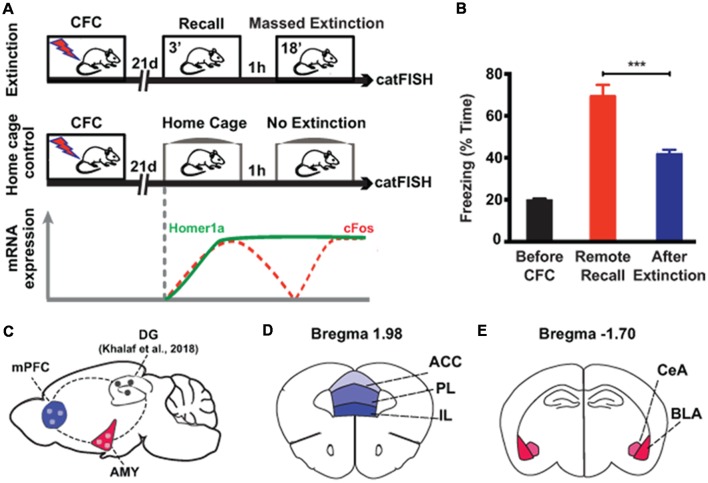
Experimental setup to study brain areas involved in remote memory attenuation by catFISH. **(A)** Schematic representation of the two experimental groups used in the study. The lower part indicates the time course of the intracellular dynamics of the RNA transcripts Homer1A and cFos, which were employed in the catFISH experiment to study brain areas engaged in remote memory recall and after the massed extinction paradigm. **(B)** Behavioral results showing the efficiency of the massed extinction paradigm to attenuate remote fear memories (*n* = 8/group, *p* < 0.001). **(C)** Schematic representation of the brain structures selected for catFISH analysis as compared to the results obtained in the dentate gyrus (DG; Khalaf et al., 2018). **(D,E)** Schematic representation of the cortical and amygdalar substructures analyzed by catFISH, respectively. CFC, contextual fear conditioning; ACC, anterior cingulate cortex; BLA, basolateral amygdala; CeA, central amygdala; IL, infralimbic cortex; PL, prelimbic cortex. ***p < 0.001, two-tailed *t*-test.

In order to investigate the cellular processes of remote fear memory attenuation in brain areas other than the hippocampus, we used a previously described massed extinction paradigm in mice (Khalaf et al., 2018; Figure 1A), which effectively reduces remote fear memories (Figure 1B, Supplementary Figure S1). This paradigm consists of the repeated exposure (6 × 3 min) of the animals to the context, which was paired with the foot shock by CFC 3 weeks earlier (Figure 1A). In parallel, we employed catFISH to harvest the intracellular spatiotemporal characteristics of different IEG mRNA species (Guzowski et al., 1999; Nonaka et al., 2014). Five minutes after the last extinction session, we identified neuronal populations activated at remote fear memory recall by the presence of cytoplasmic *Homer1a* mRNA transcripts (which appear 75 min after bouts of neuronal activity), while the neuronal populations activated by extinction were visualized with nuclear *cFos* mRNA transcripts (which remain nucleus-bound for 5 min after neuronal activity; Figure 1A). With this tool, we assessed extra-hippocampal brain areas implicated in remote memory storage, namely the amygdala and the prefrontal cortex (Kitamura et al., 2017; Figure 1C), more precisely the BLA and central amygdala (CeA; Figure 1D), as well as the anterior cingulate cortex (ACC), the PL and the IL (Figure 1E).

**Figure 2:**
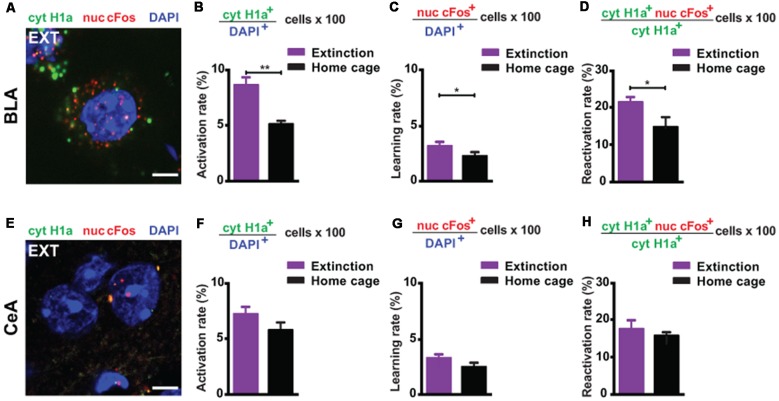
catFISH-deduced engagement of amygdala subregions upon remote fear memory recall and attenuation. **(A)** Representative image of the mRNA transcripts Homer1a and cFos in the BLA upon remote fear memory attenuation. Scale bar = 5 μm. **(B)** Homer1a-deduced activation rate in the BLA (*n* = 8–10 mice/group, *p* < 0.01). **(C)** cFos-deduced extinction learning rate in the BLA (*n* = 8–13 mice/group, *p* < 0.05). **(D)** Reactivation rate in the BLA (*n* = 8–13 mice/group, *p* < 0.05). **(E)** Representative image of the mRNA transcripts Homer1a and cFos in the CeA upon remote fear attenuation. Scale bar = 5 μm. **(F)** Homer1a-deduced activation rate in the CeA (*n* = 8–13 mice/group, n.s.). **(G)** cFos-deduced extinction learning rate in the CeA (*n* = 8–13 mice/group, n.s.). **(H)** Reactivation rate in the CeA (*n* = 8–13 mice/group, n.s.). BLA, basolateral amygdala, CeA, central amygdala. **p < 0.01, two-tailed t-test; *p < 0.05, two-tailed *t*-test.

First, we investigated the amygdala, a key limbic structure for the encoding, recall and attenuation of recent fear memories (Trouche et al., 2013; Silva et al., 2016; Tovote et al., 2016), and for the storage of remote fear memories (Maren et al., 1996; Kitamura et al., 2017; Supplementary Figure S2). For the BLA, we found a significant engagement at both memory recall (i.e., the amount of cytoplasmic *Homer1a^+^* cells normalized to the total amount of cells) and after the last extinction trial (i.e., the amount of nuclear *cFos*^+^ cells normalized to the total amount of cells) when compared to a home cage control group (Figures 1A, 2A–C). These results reflect that the BLA is activated at both time points. What is more, we found a significantly elevated reactivation rate (calculated as the amount of double positive cytoplasmic *Homer1a*^+^/nuclear *cFos*^+^ cells normalized to the total amount of cytoplasmic *Homer1a*^+^ cells) in the group that underwent massed extinction compared to the home cage control group (Figure 2D), indicating that upon remote fear attenuation a substantial proportion of the original memory trace active when behavioral expression of fear was high becomes reactivated when fear expression is low.

In the CeA (Figure 2E), neither the recall-induced activation rate (Figure 2F), nor the extinction-induced learning rate (Figure 2G), nor the reactivation rate (Figure 2H) was different between the extinction and the home cage control group. These results suggest that the CeA is not engaged upon remote memory recall, does not become activated by massed extinction and that the memory trace active at recall is not re-engaged by the extinction procedure.

**Figure 3:**
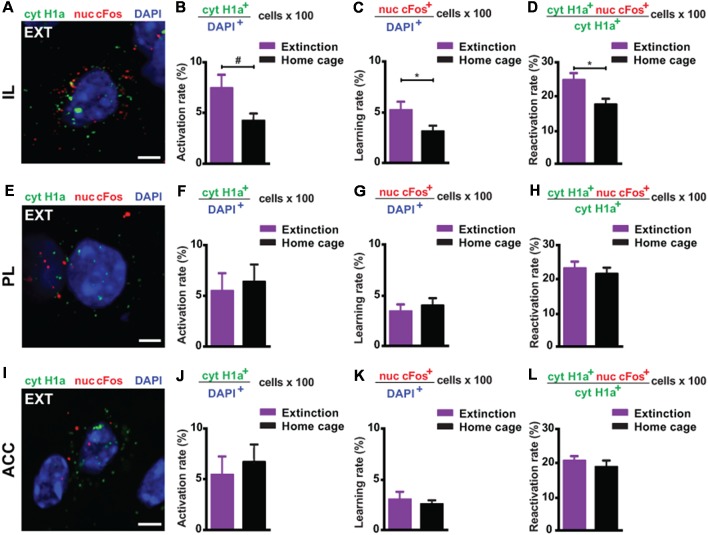
catFISH-deduced engagement of cortical subregions upon remote fear memory recall and attenuation. **(A)** Representative image of the mRNA transcripts Homer1a and cFos in the IL upon remote fear attenuation. Scale bar = 5 μm. **(B)** Homer1a-deduced activation rate in the IL (*n* = 5–9 mice/group, *p* = 0.0595). **(C)** cFos-deduced extinction learning rate in the IL (*n* = 5–6 mice/group, *p* < 0.05). **(D)** Reactivation rate in the IL (*n* = 5–6 mice/group, *p* < 0.05). **(E)** Representative image of the mRNA transcripts Homer1a and cFos in the PL upon remote fear attenuation. Scale bar = 5 μm. **(F)** Homer1a-deduced activation rate in the PL (*n* = 8–9 mice/group, n.s.). **(G)** cFos-deduced extinction learning rate in the PL (*n* = 8–9 mice/group, n.s.). **(H)** Reactivation rate in the PL (*n* = 8–9 mice/group, n.s.). **(I)** Representative image of the mRNA species Homer1a and cFos in the ACC upon remote fear attenuation. Scale bar = 5 μm. **(J)** Homer1a-deduced activation rate in the ACC (*n* = 8–9 mice/group, n.s.). **(K)** cFos-deduced extinction learning rate in the ACC (*n* = 4–8 mice/group, n.s.). **(L)** Reactivation rate in the ACC. (*n* = 4–8 mice/group, n.s.). ACC, anterior cingulate cortex; IL, infralimbic cortex; PL, prelimbic cortex. ^#^*p* < 0.06; *p < 0.05, two-tailed *t*-test.

Next, we investigated the prefrontal cortex, a crucial structure for remote memory storage (Frankland and Bontempi, 2005; Kitamura et al., 2017; Supplementary Figure S3). For the IL, we observed a strong engagement at remote memory recall (Figures 3A,B). Upon remote memory extinction, the activity of the IL was also elevated in the extinction group compared to the home cage control group (Figure 3C). Furthermore, we found a significant reactivation of recall-induced neurons by the extinction procedure (Figure 3D), indicating that a part of the original fear memory trace in the IL is still active after fear attenuation.

Conversely, in the ACC and PL, we did not find any activation at remote recall (Figures 3E,F,I,J) and after behavioral extinction (Figures 3G,K). Likewise, no reactivation was observed in either structure (Figures 3H,L). These results suggest—based on the methodology employed here—that neither the ACC nor the PL is engaged by remote fear memory recall and its attenuation.

## Discussion

Here, using catFISH of the IEGs *Homer1a* and *cFos*, we found that the BLA and IL were not only activated by remote fear memory recall and upon remote fear memory attenuation but also that a significant proportion of recall-induced neurons in these structures was reactivated when behavioral expression of fear was low. In contrast, none of these changes were observed for the CeA, the PL and the ACC (Silva et al., 2019).

This study is only the second of its kind to simultaneously study the involvement of different brain areas in remote fear memory attenuation (Silva et al., 2019), which spotlights the paucity of research conducted in this domain. Interestingly, although these studies used different IEG visualization tools (i.e., mRNA vs. protein level), different IEGs (*Homer1a* and *cFos* vs. *cFos* alone) and different paradigms of remote fear memory attenuation (massed vs. spaced extinction), both found the BLA and the IL to be engaged upon remote fear memory recall and its attenuation. These findings are also in line with a persistent implication of the BLA in fear memory storage over time (Maren et al., 1996; Goshen et al., 2011; Do-Monte et al., 2015; Kitamura et al., 2017), and thus expand the well-established role of this structure in recent fear memory attenuation (Phelps et al., 2004; Herry et al., 2010) to remote fear memories. Likewise, the results presented here further translate the importance of the IL for the attenuation of recent fear memories (Santini et al., 2008; Rosas-Vidal et al., 2014; Awad et al., 2015) to remote ones (Silva et al., 2019). In contrast, no engagement of the CeA and the ACC was detected for either remote fear recall or its attenuation, suggesting these subregions to be of minimal importance for remote fear memory attenuation. In the case of the ACC, this finding is discordant with earlier studies that had implicated the ACC in the recall of remote fear memories (Bontempi et al., 1999; Frankland et al., 2004; Goshen et al., 2011; Silva et al., 2019). Lastly, by protein-based IEG cFos studies the PL has recently been shown to be involved in both remote memory storage (Wheeler et al., 2013; Kitamura et al., 2017; Silva et al., 2019) and attenuation (Silva et al., 2019), while the current study did not reflect such a role. These discrepancies are likely due to the subtle differences in memory age, the different IEG methodologies or conditioning paradigms employed, and await functional investigations to be resolved.

In addition to being activated by remote fear memory recall and its attenuation, both the BLA and the IL showed a significantly elevated reactivation rate. At cellular resolution, these findings extend a previous report documenting a similar reactivation in another brain area, namely the DG (Khalaf et al., 2018). Given the reduced expression of fear after the massed extinction procedure, this could stipulate that the continued engagement of the original memory trace of fear in the BLA and IL is needed for fear reduction to occur, in analogy to the findings in the DG. While this interpretation remains speculative at this point and the functional experiments to address it beyond the scope of the present manuscript, several lines of evidence nevertheless point in its favor. First, by engram-specific gain and loss-of-function experiments of recall-induced neurons in the DG, a brain region that had previously been implicated in behavioral extinction (Bernier et al., 2017), such reactivation was shown to be essential for the occurrence of remote fear memory attenuation (Khalaf et al., 2018). Second, such reactivation of the fear memory engram might represent a physiological basis for learning inside the original memory trace. This renewed learning period may then serve the purpose to re-learn (or disassociate) the association formed between environmental happenstances present at encoding and the ensuing fearful response, akin to reconsolidation-updating (Morris et al., 2006). In line, empirical evidence from psychology and psychotherapy emphasizes that the recall of a trauma ought to be as complete as possible for exposure-based therapies to be effective (Foa and Kozak, 1986; Foa, 2000; Nemeroff et al., 2006), which cannot be solely explained by fear memory inhibition through extinction. Nevertheless, the participation of extinction-specific inhibitory processes cannot be ruled out with the present results, and indeed is likely given the low percentage of reactivated cells. Thus, a parsimonious explanation for the cellular processes underlying remote fear memory attenuation combines both extinction-specific, inhibitory processes mediated by a newly learned memory trace of safety that is different from the original trace of fear, together with a reconsolidation-updating process that is mediated by a re-learning of the original memory trace of fear towards safety, which occurs in recall-induced neurons.

Future studies should thus be aimed at further disentangling extinction from reconsolidation-updating processes, for example by addressing the causal implication of recall-induced neurons in memory attenuation, or by addressing the behavioral consequence of functionally manipulating remote extinction-induced neurons. Anatomically, this is of special interest for the two key brain areas emanating from the present study, the BLA and the IL, as the IL is known to send monosynaptic inhibitory projections to the BLA that are important for the attenuation of recent fear memories (Herry et al., 2008, 2010; Awad et al., 2015) and to store memories of past extinction trials (Milad and Quirk, 2002). In the BLA, it would further be interesting to decipher whether the reactivated neurons are those responsible for creating negative or positive associations (Namburi et al., 2015), and how they dynamically develop over the course of extinction (Grewe et al., 2017). This is of particular relevance for long-lasting memories since the vast majority of findings concerning extinction and reconsolidation-updating have been obtained by studying day-old fear memories, despite the fact that traumatic memories are extremely persistent and can impinge on one’s emotional well-being for a long time after the trauma. As remote fear memories are stored differently than recent ones (Frankland and Bontempi, 2005; Frankland et al., 2006; Khalaf and Gräff, 2016; Kitamura et al., 2017; Albo and Gräff, 2018; Tonegawa et al., 2018) and appear to more difficult to attenuate (Milekic and Alberini, 2002; Costanzi et al., 2011; Gräff et al., 2014; Tsai and Gräff, 2014), this represents a fundamental gap in memory research.

In sum, this study shows that recall-induced neurons in both the BLA and IL become reactivated upon remote fear memory attenuation, which extends previous findings from the DG (Khalaf et al., 2018). Together, these results indicate that an active participation of the original fear trace towards fear memory attenuation may be a conserved mechanism across brain areas that are engaged by remote fear memory recall.

## Author Contributions

OK and JG designed the experiments and wrote the article. OK carried out the experiments and analyzed the data.

## Conflict of Interest Statement

The authors declare that the research was conducted in the absence of any commercial or financial relationships that could be construed as a potential conflict of interest.
