# Supplementary material for: Reactivation of Recall-Induced Neurons in the Infralimbic Cortex and the Basolateral Amygdala After Remote Fear Memory Attenuation
Source: Front Mol Neurosci. 2019 Apr 17;12:70. doi: 10.3389/fnmol.2019.00070 (PMC6481183; doi:10.3389/fnmol.2019.00070)
Supplement: Supplementary file 1 [file Data_Sheet_1.PDF]

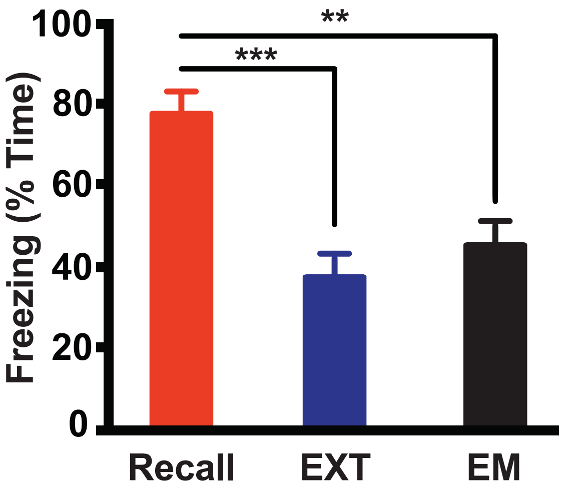

Supplementary Figure 1 I The massed extinction paradigm persistently attenuates remote fear memory. Behavioral results showing the efficiency of the massed extinction paradigm to attenuate remote fear memories. EXT, massed extinction; EM, extinction memory. Repeated measurement ANOVA, $p<0.001$, followed by Tukey post hoc test ($n= 10$, ***$p<0.0001$, **$p<0.001$).

Khalaf and Gräff, Supp. Fig. 1

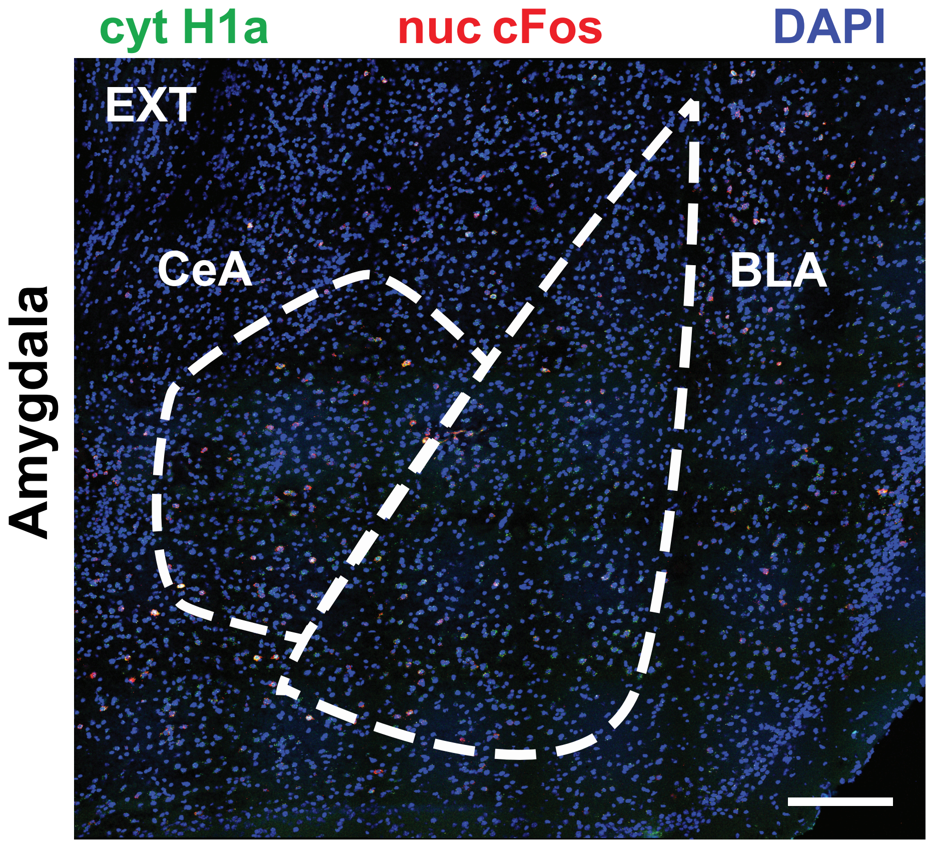

Supplementary Figure 2 I Low-resolution image of the catFISH labeling in the amygdala. Scale bar=50µm.

Khalaf and Gräff, Supp. Fig. 2

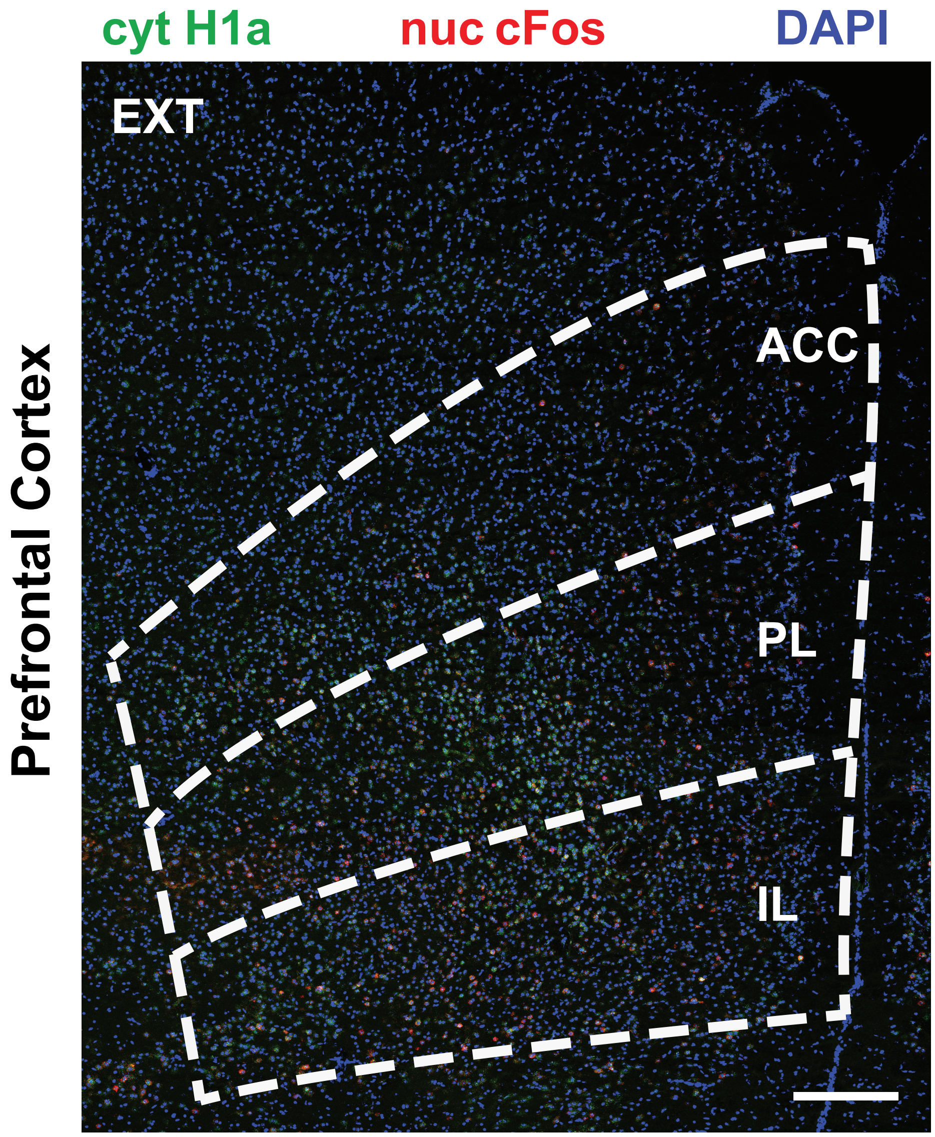

Supplementary Figure 3 I Low-resolution image of the catFISH labeling in the prefrontal cortex. Scale bar=50µm.
